# Drug utilization in patients starting haemodialysis with a focus on cardiovascular and antidiabetic medications: an epidemiological study in the Lazio region (Italy), 2016–2020

**DOI:** 10.1186/s12882-024-03539-5

**Published:** 2024-03-16

**Authors:** Ursula Kirchmayer, Claudia Marino, Sandro Feriozzi, Carlo Massimetti, Micol Manzuoli, Laura Angelici, Anna Maria Bargagli, Silvia Cascini, Antonio Addis, Marina Davoli, Nera Agabiti

**Affiliations:** 1 Department of Epidemiology ASL Roma 1, Lazio Regional Health Service, Via Cristoforo Colombo 112, 00147 Rome, Italy; 2 UOC Nephrology and Dialysis, ASL Viterbo, Viterbo, Italy

**Keywords:** Dialysis, Drug therapy, Diabetes, Cardiovascular, Observational study

## Abstract

**Background:**

Entering dialysis is a critical moment in patients’ healthcare journey, and little is known about drug therapy around it. A study funded by the Italian Medicines Agency offered the opportunity to leverage data from the Lazio Regional Dialysis and Transplant Registry (RRDTL) and perform an observational study on drug use patterns before and after initiating chronic dialysis.

**Methods:**

Individuals initiating dialysis in 2016–2020 were identified from RRDTL, excluding patients with prior renal transplantation, stopping dialysis early, or dying within 12 months. Use of study drugs, predefined by clinicians, in the two years around the index date was retrieved from the drug claims register and described by semester. For each drug group, proportions of users (min 2 claims in 6 months) by semester, and intensity of treatment in terms of Defined Daily Doses (DDDs) for cardiovascular and antidiabetic agents were compared across semesters, stratifying by sex and age.

**Results:**

In our cohort of 3,882 patients we observed a general increase in drug use after initiating dialysis, with the mean number rising from 5.5 to 6.2. Cardiovascular agents accounted for the highest proportions, along with proton pump inhibitors and antithrombotics over all semesters. Dialysis-specific therapies showed the most evident increase, in particular anti-anaemics (iron 4-fold, erythropoietins almost 2-fold), anti-parathyroids (6-fold), and chelating agents (4-fold). Use of cardiovascular and antidiabetic drugs was characterised by significant variations in terms of patterns and intensity, with some differences between sexes and age groups.

**Conclusions:**

Entering dialysis is associated with increased use of specific drugs and goes along with adaptations of chronic therapies.

**Supplementary Information:**

The online version contains supplementary material available at 10.1186/s12882-024-03539-5.

## Background

Patients needing chronic dialysis are frail and in addition to having end-stage chronic kidney diseasemost are affected by other chronic conditions, including anaemia, renal bone disease, cardiovascular diseases and diabetes [1–3]. According to the Lazio Regional Dialysis Register (RRDTL) annual report, almost 75% of dialysis patients suffer from at least one comorbidity, with the highest proportions for cardiovascular diseases and diabetes [4].

Diabetes is associated with diabetic kidney disease, inducing kidney inflammation and fibrosis through the metabolic pathway [5] and is one of the leading causes of end-stage renal disease [6]. A systematic review of observational studies reported estimates of the annual incidence of microalbuminuria and albuminuria for Type 2 diabetes ranging from 3.8 to 12.7% and end-stage renal disease between 0.04% and 1.8% [7]. Diabetes management in patients entering dialysis is challenging and requests special attention regarding the choice of antidiabetic therapy and adjustments in the diabetes medication regimen [8].

Cardiovascular diseases are strongly associated with chronic renal disease and progressive impairment of the glomerular filtration rate. This is due to increased arterial hypertension, anaemia, disorders of calcium-phosphorus homeostasis and diabetes mellitus occurring in chronic renal failure. The risk of death in the uremic population due to cardiovascular complications is particularly high. Therefore, the treatment of cardiovascular complications is crucial in reducing mortality in dialysis units [9–11]. Analyzing drug treatment patterns in the dialytic population provides valuable information for caregivers and policymakers to enable clinical audits for potentially inappropriate prescribing, medication review and health burden [12].

In Italy and in the Lazio region, a comprehensive overview of drug use in the dialysis populations is not available to date. The regional pharmacovigilance project on evaluating the effectiveness and safety of etelcalcetide in treating secondary hyperparathyroidism in patients undergoing haemodialysis offers the opportunity to systematically investigate the use of specific drugs in this population and compare the changes in use patterns before and after starting dialysis.

Uptake and increase in specific therapies, such as treatments for anaemia, renal bone disease, or electrolyte balancing agents, are expected in patients entering dialysis. At the same time, less is known about adaptations of other chronic medications in this critical clinical phase. Therefore, we focused on those medications, which are not specific for chronic disease, but for treating the most vital co-existing conditions, i.e., cardiovascular diseases and diabetes, which might require modifications in terms of use patterns and treatment intensity.

The study investigated how the start of haemodialysis affects the therapeutic regime in uremic patients. The beginning of haemodialysis is associated with control of uremic status; on the other hand, it exposes the patient to medical staff. In addition, many drugs can be administered during or at the end of treatment.

The present study aimed at performing a descriptive analysis on drug use in a haemodialysis population. Specifically, we compared drug use patterns in a cohort of haemodialytic patients before and after starting dialysis with a specific focus on cardiovascular and antidiabetic drugs and investigating differences related to age and gender.

## Methods

### Data sources

The present study takes advantage of the health information systems of the Lazio region. Lazio is a central region of Italy which includes the municipality of Rome. In 2022 there were about 5,7 Million residents, 48% male [13]. In particular, data were retrieved from hospital discharge records (ICD-9-CM coding for up to six diagnoses at discharge), the outpatient drug claims register (ATC classification), the mortality register, and the healthcare assistance file (enrolment in the regional healthcare system). All databases comprise individual patient records, which can be linked between databanks through a unique pseudo-anonymous patient identifier in line with current privacy legislation. Moreover, data from the RRDTL were used for the enrolment of the study population.

### Study population

From RRDTL, we enrolled all patients with incident chronic dialysis in the Lazio region from 2016 to 2020. Specialized outpatient clinics issue dialysis and all patients are in charge of a nephrologist once entering dialysis. Incident chronic dialysis was defined as starting dialysis for the first time and still being on dialysis three months after the first dialysis treatment. The date of the first dialysis treatment was defined as the index date.

We excluded patients younger than 18 years at first dialysis, those not enrolled in the regional healthcare assistance or not resident in the Lazio region, who had undergone renal transplantation before the index date or died during the 12 months after starting dialysis. This last criterion was crucial to attribute the same observation time to all patients and allow for comparison of proportions and mean Defined Daily Doses (DDDs) across semesters. We used the official WHO DDD methodology [14] which is broadly applied in pharmacoepidemiology and is an internationally recognised standard to measure and compare drug use. Patients were characterized with respect to sex, age, area of residence, and comorbidities, retrieved from hospital discharge records in the two-year look-back period before the index date, considering any of the six discharge diagnoses reported for each record. Related ICD-9-CM codes are written in Additional File 1.

### Drug use patterns

From the regional drug claims register, the use of a list of drugs typically prescribed in this population and predefined by nephrologists (Additional File 2) was retrieved at the individual level for all patients. Only drugs reimbursed by the regional healthcare service are traceable and were included. For the focus on diabetes and cardiovascular disease, drugs were grouped according to the ATC classification, accounting for group A10 drugs used in diabetes and C cardiovascular system [14]. 

Use was defined as at least two claims of a drug/drugs group in each of the four semesters around dialysis, namely months 12 − 7 before dialysis (semester 1), months 6 − 0 before dialysis (semester 2), months 0–6 after starting dialysis (semester 3), and months 7–12 after starting dialysis (semester 4).

### Statistical analyses

#### Drug use measures

Drug use was measured through proportions of users of the different drugs and intensity of drug use. This was calculated by summing up all DDDs of a drug/drug group claimed by users during each semester and calculating DDD mean values, median values, and Inter Quartile Range (IQR).

Differences in proportions and DDD mean values before and after entering dialysis were investigated, focusing on the comparison between the third and the first semester and applying chi-square tests for the proportions and Fisher’s t-test for DDD mean values.

#### Stratification by sex and age

To account for potential differences between men and women and older ages, analyses stratified by sex and age (18–64 years vs. 65 + years) were performed and are reported for the proportions of users and intensity for antidiabetic drugs only, as an example.

All data were analysed using SAS version 9.4 (SAS Institute, Cary, NC, USA).

## Results

### Characteristics of the study population

**Fig. 1:**
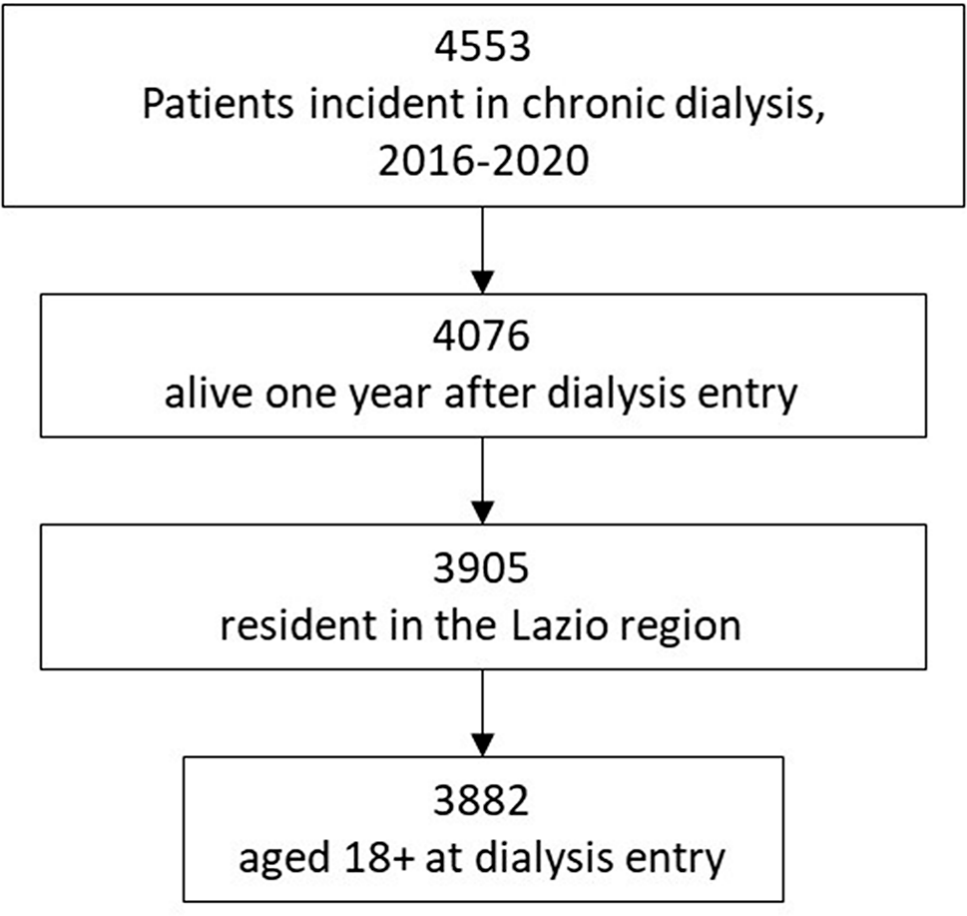
Attrition diagram of the cohort selection

Overall, 3,882 patients newly entering chronic haemodialysis in 2016–2020 were enrolled in our study (Fig. 1; Table 1, Additional File 3). Two-thirds of the cohort are men; over 63% were 65 years or older at first dialysis.

**Table 1:** Characteristics of the study population at the start of haemodialysis

	**Total**	
	N	%
Total	3882	
**Sex**		
Male	2588	*66.7*
Female	1294	*33.3*
**Age**		
18–64 years	1459	*37.6*
65 + years	2423	*62.4*
**Comorbidities**		
Type 2 diabetes	624	*16.1*
Hypertensive disease	1390	35.8
Anaemias (excl. acute post-haemorrhagic)	894	23
Ischemic heart disease	502	12.9
Heart failure	422	10.9
Cerebrovascular disease	384	9.9
Conduction disorders, cardiac dysrhythmias	313	8.1
Malignancies	299	7.7
Chronic obstructive pulmonary disease	204	5.3
Respiratory failure	179	4.6
Disorders of lipoid metabolism	160	4.1
Disorders of the thyroid gland	142	3.7
Overweight, obesity and other hyperalimentation (BMI > 30)	88	2.3
Chronic liver disease, diseases of the pancreas	87	2.2
Peptic ulcer	23	0.6
Dementias	18	0.5
Mental disorders	19	0.5
Nutritional deficiencies	16	0.4
Chronic inflammatory intestinal diseases	14	0.4

In general, the dialytic population is affected by numerous comorbidities. Patients suffering from type-2 diabetes account for 16.1%. Among the other conditions, hypertension is the most frequent one, in over 35.8% of the population, followed by anaemia (23.0%), ischemic heart disease (12.9%) and heart failure (10.9%).

### Proportions of drug users

Use of the study drugs/drug groups generally increases across the four semesters, especially in the pre-dialysis period and the first semester after dialysis initiation, with mean values (STD) of 5.54 (3.35) in semester 1, 6.24 (3.37) in semester 2, 7.30 (2.84) in semester 3, and 6.25 (3.19) in semester 4.

**Fig. 2:**
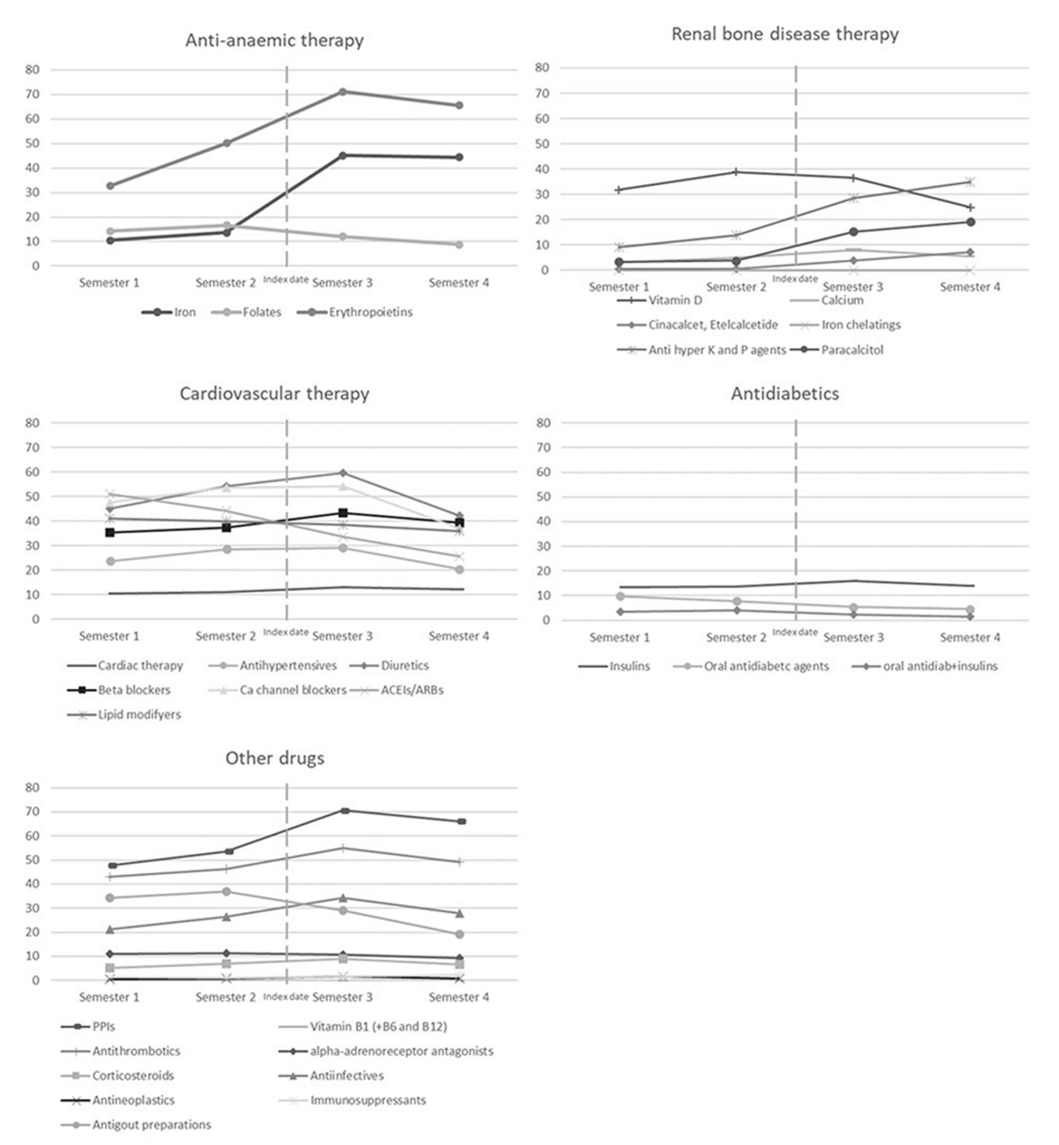
Proportions of haemodialysis patients using study drugs in the four semesters around dialysis start

Fig 2 shows the proportions of patients treated with drugs typically prescribed to the dialytic population in the four semesters around the start of dialysis. Drug therapy is frequent in our cohort and reflects the reported comorbidities. Cardiovascular therapy is the most prescribed group, along with antithrombotics and proton pump inhibitors (PPIs), followed by drugs for anaemia.

For some drug classes, differences between semesters are observed. In particular, anti-anaemic therapy with iron and erythropoietin increases steeply in the six months before and after starting dialysis (iron: from 10.6 to 44.4%, erythropoietin: from 32.6 to 65.6%). Similar trends are observed for treatments for hyperkalaemia and hyperphosphatemia (from 9.0 to 35.0%) and anti-parathyroid agents (paricalcitol from 3.4 to 19.1% and cinacalcet/etelcalcetide from 0.5 to 7.2%).

Cardiovascular therapy maintains high levels in all semesters. The choice of the different drug classes changes after dialysis, with a sharp decrease in ACEIs/ARBS (from 51.1 to 25.6%) and a temporal increase in diuretics and Calcium-channel blockers, which peaks in the first semester after initiating dialysis and then drops below initial levels in the last semester (diuretics: 45.1%, 54.1%, 59.6%, 42.1%, Calcium-channel blockers: 47.55, 53.5%, 54.1%, 37.0%). The proportion of patients prescribed antithrombotics increases, peaking in semester 3 (43.0%, 46.2%, 55.1%, 49.3%).

The use of antidiabetic drugs is basically stable over time, but changes in the patterns of specific drug utilization are observed. Prescription of oral antidiabetics and combined therapy decreased (from 9.8 to 4.5% and from 3.5 to 1.4%, respectively) while the proportion of patients using insulin alone remained relatively stable over time, with an increase immediately after starting dialysis (13.5%, 13.7%, 16.0%, 13.9%).

Among the remaining drugs, an increase in use of PPIs (from 47.7 to 66.1%) and anti-infectives (from 21.3 to 27.9%), and a decrease in reducing plasma acid uric medicines is observed (from 34.3 to 19.1%), while proportions of use of all other agents remain stable.

Differences before and after entering dialysis were statistically significant for almost all drugs/drug classes, except for Insulin, Vitamin D, and Iron chelating agents/Drugs for the treatment of hyperkalaemia and hyperphosphatemia.

**Fig. 3:**
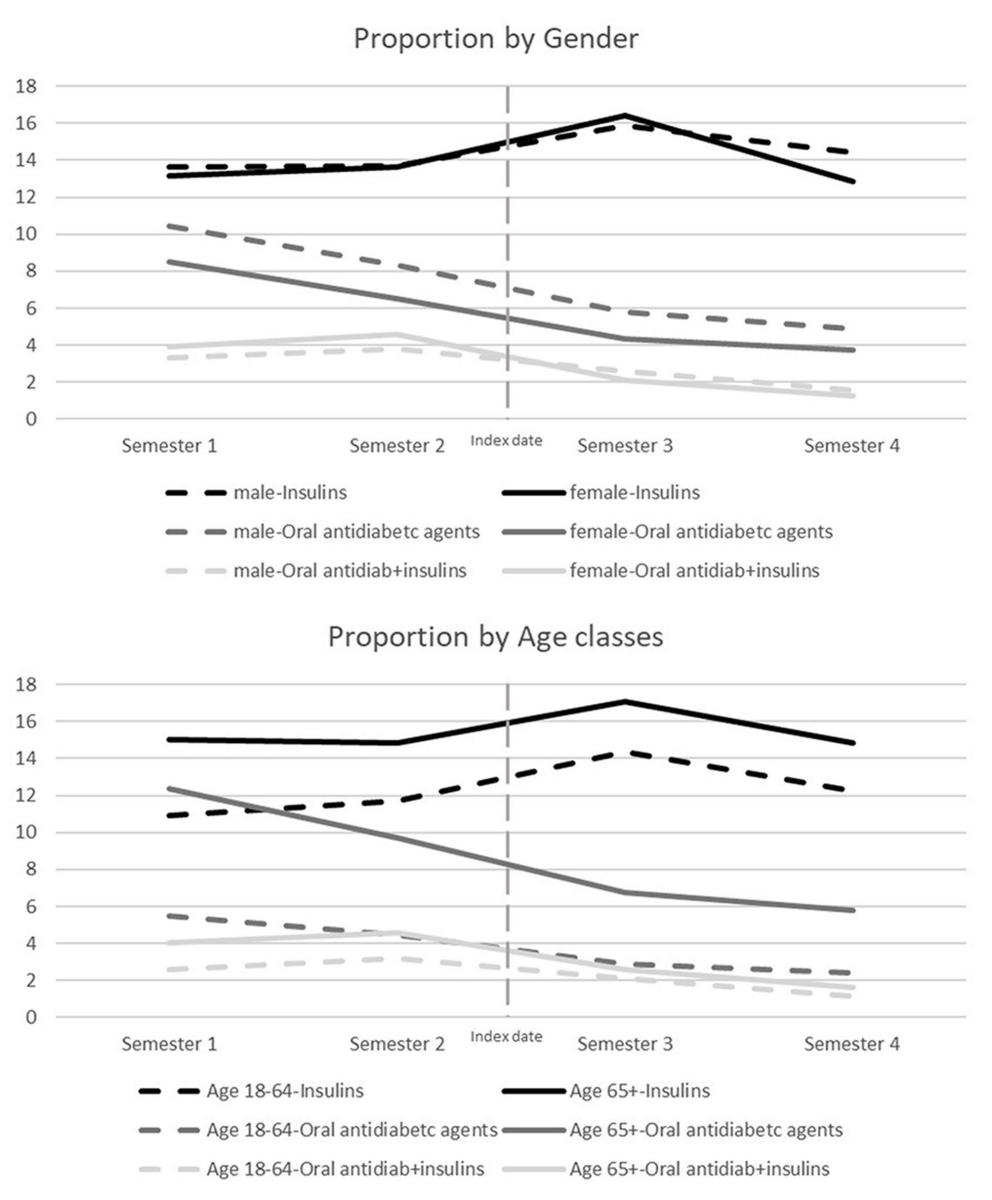
Proportions of patients using antidiabetics in the semesters around dialysis start by sex and age

Results of the stratified analyses for antidiabetic drugs are shown in Fig. 3, and for all other drugs in the additional files (Additional files 4 and 5). Proportions of patients treated with oral antidiabetics alone are generally higher in men than in women. For insulin alone, a slight increase is observed in men (from 13.6% in semester 2 to 14.4% in semester 4), whereas in women, this is evident only immediately after the index date. The proportions of older patients treated with antidiabetics are higher than in the younger group. The decrease for oral formulations is steeper in older respect to younger patients (from 12.4 to 5.8% vs. from 5.5 to 2.4%). In contrast, for insulins, the proportion of younger users remains higher in the last semester than before (12.3% previous semester vs. 10.9% first semester), which is not the case in the older (14.9% vs. 15.0%).

### The intensity of treatment use in drug users

**Fig. 4:**
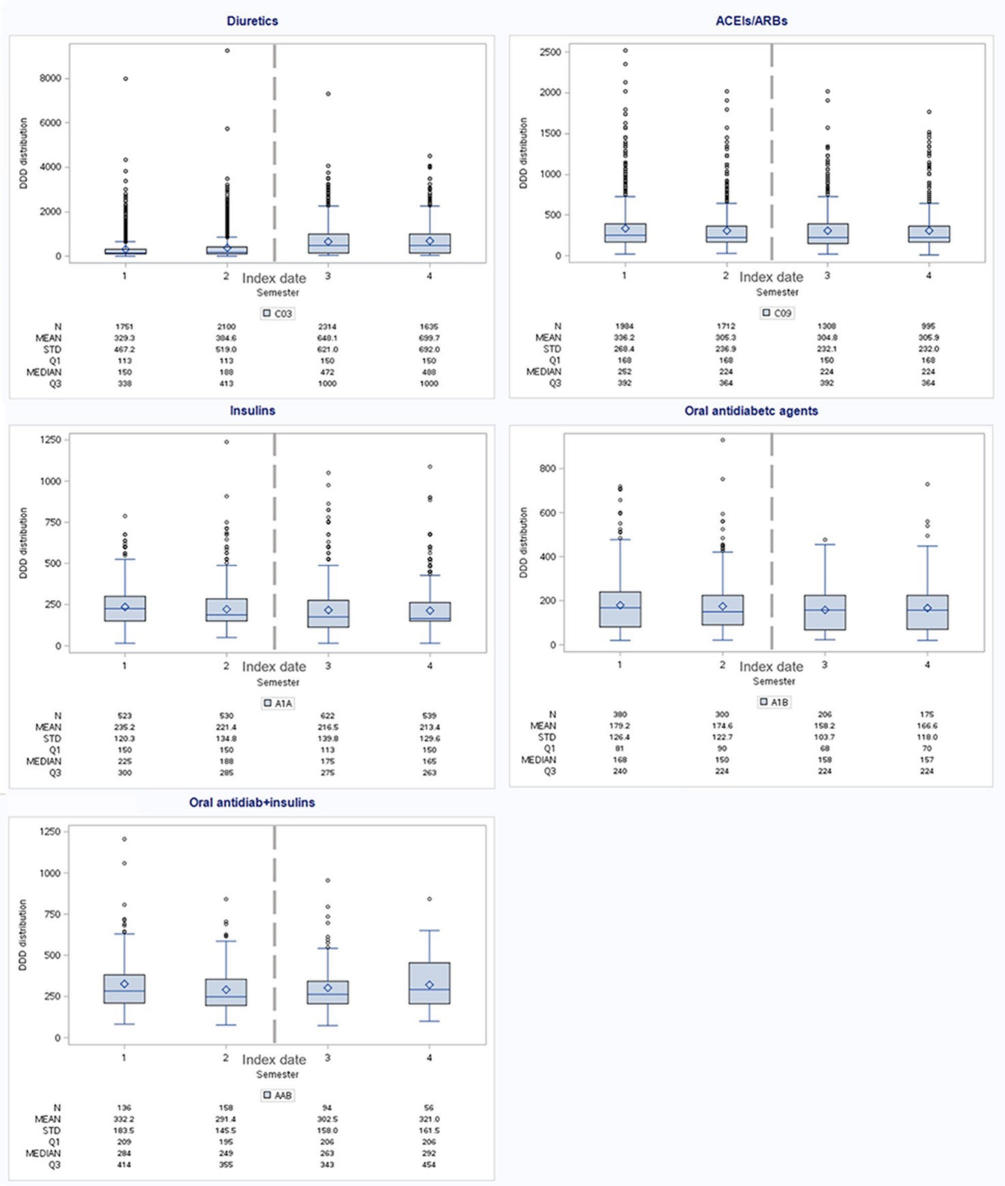
Intensity of therapy with diuretics, ACEIs/ARBs, and antidiabetics in the four semesters

Results of the analysis of treatment intensity among users of selected cardiovascular and antidiabetic medications over the four semesters in terms of DDDs (mean and median values, as well as IQRs) are shown in Fig. 4 and in the additional file 6 for other cardiovascular drugs.

The essential treatment adaptations were detected for diuretics: mean doses of diuretics increased sharply in the semester before and the two semesters after dialysis initiation in patients treated, and over time more than doubled (mean DDDs from 329.3 to 699.7), associated with increased variability (IQR _semester 2_ vs. IQR _semester 4_: from 113 to 338 to 150–1000).

For ACEIs/ARBs, reductions in mean DDDs were detected in the semester immediately before starting dialysis (from 336.2 to 305.3 mean DDDs), and levels remained stable after that.

Regarding antidiabetic therapy, doses are steadily reduced over time for insulins (from 235.2 to 213.4 mean DDDs), whereas variable trends are found for oral antidiabetics (179.2–174.6–158.2–166.6 mean DDDs) and combined therapy (332.2–291.4–302.5–321.0 mean DDDs).

These differences were all statistically significant.

**Fig. 5:**
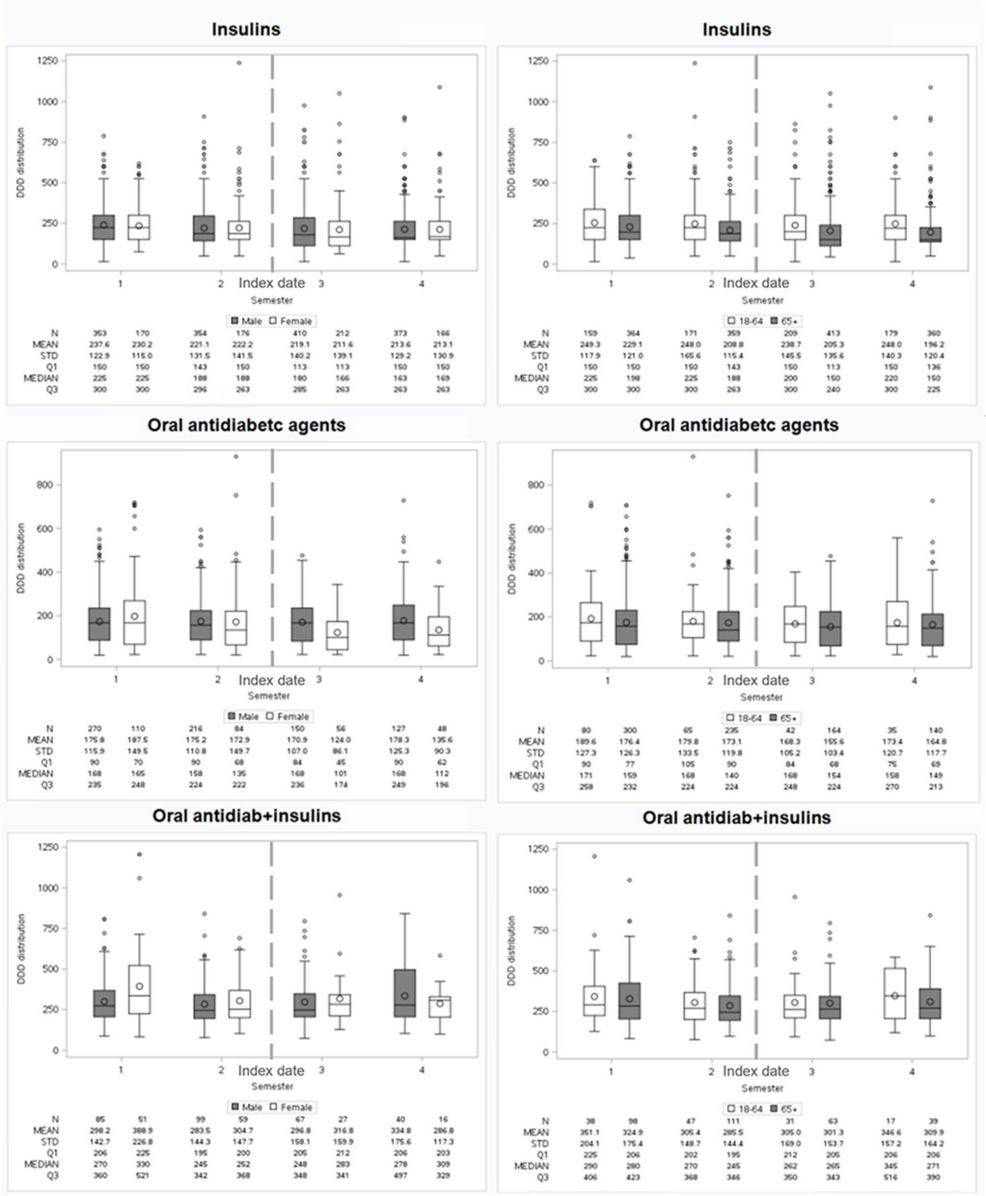
Intensity of antidiabetic therapy in the four semesters by sex and age

Stratifying by sex (Fig. 5), no differences are found for insulins, with similar mean DDDs decreasing over the semesters for both sexes. Mean doses of oral formulations are higher in women in the first semester, but due to a steady reduction in women along with stable doses in men, in the last semester, mean DDDs are higher in men. For the combined therapy, doses increase in men and decrease in women.

Age-specific analyses (Fig. 5) show higher mean DDDs for the younger age group for all antidiabetic formulations. Insulin doses remain stable in 18–64-year-old patients. At the same time, they decrease in the 65 + age group, doses of oral drugs are reduced in all ages, and the combined therapy shows an initial decrease followed by an increase 1 year after dialysis initiation.

In general, for cardiovascular therapy, dose adaptations around the initiation of dialysis are observed for all agents (Additional File 6). For almost all cardiovascular agents, mean doses are higher in men, except for diuretics and beta blockers (Additional File 7). Younger patients are prescribed higher doses except for lipid modifiers and antithrombotics, with similar doses between age groups (Additional File 8).

## Discussion

The present study draws a picture of the pharmacological therapy typically prescribed to dialytic patients in a large cohort, comparing patterns in the year before and after initiation of dialysis and for antidiabetic medications also stratified by sex and age.

The sociodemographic characteristics of our cohort reflect those typically described for patients in dialysis in terms of age and sex distributions in our region [4].

Concerning pharmacotherapy, our findings align with the comorbidities we detected for the included patients and clinical characteristics previously reported for patients in the same registry [4]. The high proportions of patients using drugs for cardiovascular diseases, diabetes, and gout are plausible because these comorbidities may affect the clinical course of kidney disease [6].

We observed an increase in use of specific drugs, typically indicated in dialysis, namely therapies for anaemia and renal bone disease, as expected in patients initiating dialysis [15].

These changes are basically due to the closer medical control the patient starting dialysis usually undergoes. Clinical and laboratory tests are more frequent, and continuous progressive adjustment of drugs therapy occurs. Frequently checked in the first weeks of dialysis, haemoglobin values lead to erythropoietin-stimulating agents and iron prescription.

The start of dialysis significantly impacts secondary hyperparathyroidism, and the therapy is usually changed. We observed an increase of drugs to modulate the parathyroid hormone (paricalcitol and etelcalcetide/cinacalcet). There was less use of vitamin D, which likely correlated with the effect of parathyroid modulation. The phosphorous binders were prescribed because the phosphorous dialytic clearance was low.

We observed increased prescriptions for cardiovascular medications in the early phase of dialysis, followed by a substantial reduction and a final plateau. It is interesting to note the early rise in diuretics likely to maintain a residual diuresis with a following reduction and the progressive reduction of the use of drugs interfering with the renin-angiotensin-aldosterone axis (ACEIs and ARBs). The decline could be associated with the side effect of hyperkalaemia. Consequently, there is also an increase in medications to control serum potassium levels. The reduction of ACEIs/ARBs can be criticized according to the guidelines criteria. Indeed, these drugs are recommended for multiple actions on cardiac function (blood pressure, cardiac failure, tissue fibrosis etc.), although the combination of ACEIs/ARBs is not recommended [16]. It seems evident that the worry about life-threatening hyperkalaemia events exceeds the positive expectations associated with these drugs. However, the availability of new drugs to control hyperkalaemia, such as patiromer and sodium zirconium cyclosilicate, should encourage the use of ACEIs or ARBs.

Patients who have type-2 diabetes are at risk of diabetic kidney diseases; consequently, diabetes is one of the comorbidities most commonly found in this population. Accordingly, treatment with antidiabetic medications was observed in many patients. Diabetes management in patients with renal impairment is challenging. Active agents, frequently used before dialysis, such as metformin, are not recommended or poorly used once the patient enters dialysis [17]. Consequently, antidiabetic therapy needs to be adapted after starting dialysis, and changes have been detected in our study, both in prescription patterns and treatment intensity. The observed time trends in the four semesters around dialysis initiation reflect therapeutic adaptations in the most critical phase of patient management, with modifications starting in the six months preceding dialysis and the most evident variations immediately after dialysis initiation. This might be partly because starting dialysis is associated with changes in care, i.e., for patients accessing dialysis centres, the entire clinical situation is newly evaluated and re-balanced by specialists.

The shares of patients treated with insulin, oral antidiabetics or the combination align with results from a large cohort of patients with chronic kidney disease [17]. The differences observed between men and women and between younger and older patients align with the prevalence of diabetes in this population.

The increased use of drugs with the start of dialysis could indicate that the physicians’ attention in the last part of chronic renal failure is paid to an appropriate timing for the replacement therapy more than closely managing clinical and laboratory issues. Moreover, the start of dialysis is associated with more frequent patient access to the nephrology centre, facilitating the medical control and the prescription and administration of drugs, during dialysis treatment. It is unclear if this behaviour can influence the outcome of the patients. From our data, we cannot infer how this approach can affect the outcome of uremic patients, especially in the first period of dialysis when the mortality rate is higher due to many reasons [18]..

Our data show that starting dialysis is associated with increased medication use. Moreover, quality of life does not constantly improve in these patients due to the changes in daily habits, familiar dynamics and side effects of the dialysis treatment. This strengthens the need to appropriately manage advanced chronic renal failure to limit the related adverse impacts. Indeed, the start of dialysis therapy determined changes in the quality of life that confound the medical effect of these new therapeutic regimens. The correction of uremic dialysis and better control of clinical parameters improve the life prognosis of patients, and the drug effect is not discernible from the dialytic effect.

To our knowledge, this is the first epidemiological study systematically evaluating patterns of drug therapy in a population-based dialytic cohort in Italy. The availability of a data from the regional dialysis registry allowed enrolment of clinically confirmed dialytic patients and the definition of the exact date of dialysis initiation for each patient. Pharmacotherapy was retrieved from the regional outpatient drug claims register, which covers all drugs refunded by the regional public health service, including the chronic treatments we focused on. Our focus was on two major drug groups, antidiabetics and cardiovascular drugs because cardiovascular disease and diabetes represent conditions strongly linked with chronic kidney disease in terms of etiopathogenetic and have a high prevalence in our cohort.

There are also some limitations inherent to the nature of administrative healthcare data. For example, the retrieval of comorbidities is based on hospital discharge diagnoses, which sometimes does not capture all patients affected by a disease, especially if the condition does not request hospitalization, such as diabetes. Whereas our hospital discharge data found 16% of the cohort with diabetes, RRDTL data for the same patients yield 29%. Using drug claims data bares the risk of underestimation due to the impossibility of tracing over-the-counter drugs or purchased paying out-of-pocket (e.g. iron chelating agents), even if in a population with intense chronic treatment and frequent medical contact, this is expected to account for a tiny proportion. Moreover, claiming a drug is a proxy for drug consumption, but stocking medications in patients’ homes is frequent. This might mainly affect the intensity analysis, and consumption may be overestimated. Also, our data do not provide information on individual doses, and using the DDDs as a standard dose may be inaccurate, e.g., insulins. Yet, while over- or underestimation may impact proportions and intensity estimates, they do not alter the comparison of the estimates between periods, which was the aim of this study, and the use of the drugs of interest was also confirmed by data available from RRDTL. The present study aimed to draw an overall picture of use patterns while investigating the use of different active agents within a drug class, such as for diuretics or antithrombotics, which was not part of our objectives but might be analyzed in terms of appropriateness in future studies.

## Conclusion

Patients entering dialysis are a frail population and are often affected by comorbidities. Consequently, the proportions of chronic drug therapies are high.

Starting dialysis is a critical phase in patients with renal impairment. The pre-existing pharmacological therapy is often integrated with specific medicines, such as anti-anaemic (iron and erythropoietin), treatments for hyperkalaemia and hyperphosphatemia, and anti-parathyroid agents, particularly paricalcitol.

Chronic therapies prescribed before dialysis are usually continued also after entering dialysis, but the choice of active agents may change. Starting dialysis shifts therapies from oral antidiabetics alone or a combination of insulin plus oral antidiabetics towards treatment with insulin. Regarding cardiovascular drugs, dialysis is associated with increased use of diuretics, especially in the transition period around imitation of dialysis, while cardiovascular other medicines are often reduced.

This scenario has potential strong implications in terms of health care costs.

In summary, prescribers need to pay special attention to patients entering dialysis, and our study’s findings offer information that might be useful for clinical audit and feedback.

### Electronic supplementary material

Below is the link to the electronic supplementary material.

Supplementary Material 1

Supplementary Material 2

Supplementary Material 3

Supplementary Material 4

Supplementary Material 5

Supplementary Material 6

Supplementary Material 7

Supplementary Material 8

## Data Availability

Data related to the findings reported in our manuscript are available to all interested researchers upon request because of stringent legal restrictions regarding privacy policy on personal information in Italy (national legislative decree on privacy policy n. 196/30 June 2003). For these reasons, our dataset cannot be made available on public data deposition. All interested researchers can contact the following persons to request the data: Nera Agabiti, Department of Epidemiology, Lazio Regional Health Service, Rome, Italy, e-mail: n.agabiti@deplazio.it; Damiano Lanzi, Department of Epidemiology, Lazio Regional Health Service, Rome, Italy, e-mail: d.lanzi@deplazio.it.

## Abbreviations

ACEI: Angiotensin-Converting Enzyme Inhibitors
ARB: Angiotensin II receptor blocker
ATC: Anatomical Therapeutic Chemical Classification
BMI: Body Mass Index
DDD: Defined Daily Dose
ICD-9-CM: International Classification of Disease, Nine Revision, Clinical Modification
IQR: Interquartile Range
PPI: Proton Pump Inhibitor
RRDTL: Lazio Regional Dialysis and Transplant Registry
STD: Standard Deviation
