# Supplementary material for: Drug utilization in patients starting haemodialysis with a focus on cardiovascular and antidiabetic medications: an epidemiological study in the Lazio region (Italy), 2016–2020
Source: BMC Nephrol. 2024 Mar 16;25:98. doi: 10.1186/s12882-024-03539-5 (PMC10943891; doi:10.1186/s12882-024-03539-5)
Supplement: Supplementary file 1 — Supplementary Material 1 [file 12882_2024_3539_MOESM1_ESM.docx]

Additional file 1. Comorbidities

| **Condition** | **ICD-9-CM codes** |
| --- | --- |
| Malignancies | 140–208, V10 |
| Disorders of thyroid gland | 240-246, 252, 6.8 (procedure) |
| Disorders of lipoid metabolism | 272 |
| Nutritional deficiencies | 260-263 |
| Overweight, obesity and other hyperalimentation (BMI>30) | 278, V85.3, V85.4 |
| Anaemias (excl. acute post haemorrhagic) | 280-284, 285 (excl. 285.1) |
| Dementias | 290.0-290.4, 294.1, 331.0 |
| Mental disorders | 293.8, 295-298, 299.1, 300.4, 301.12, 309.0, 309.1, 311 |
| Hypertensive disease | 401-405 |
| Ischemic heart disease | 410, 412, 411, 413, 414 429.7 |
| Conduction disorders, cardiac dysrhythmias | 426, 427 |
| Heart failure | 428 |
| Cerebrovascular disease | 430-432, 433, 434, 436, 437, 438, 435, 440-448, 557 |
| Chronic obstructive pulmonary disease | 490-496 |
| Respiratory failure | 518.81, 518.82, 518.83, 518.84 |
| Chronic liver disease, diseases of pancreas | 571-573, 577 |
| Peptic ulcer | 530.2, 531-534 |
| Chronic inflammatory intestinal diseases | 555-556 |
| Diabetes mellitus | Validated algorhythm (ref) |
