# Supplementary material for: Drug utilization in patients starting haemodialysis with a focus on cardiovascular and antidiabetic medications: an epidemiological study in the Lazio region (Italy), 2016–2020
Source: BMC Nephrol. 2024 Mar 16;25:98. doi: 10.1186/s12882-024-03539-5 (PMC10943891; doi:10.1186/s12882-024-03539-5)
Supplement: Supplementary file 2 — Supplementary Material 2 [file 12882_2024_3539_MOESM2_ESM.docx]

Additional file 2. List of drugs of interest in dialytic patients

| **Chronic condition** | **Drug/drug group** | **ATC code** |
| --- | --- | --- |
| **Anaemia** | Iron preparations | B03A |
|  | Folic acid and derivatives | B03BB |
|  | Other anti-anaemic preparations (erythropoietins) | B03XA |
| **Renal Bone Disease** | Vitamin D and analogues | A11CC |
|  | Calcium | A12AA |
|  | Paricalcitol, Cinacalcet, Etelcalcetide | H05BX |
|  | Iron chelating agents | V03AC |
|  | Drugs for treatment of hyperkalaemia and hyperphosphatemia | V03AE |
| **Cardiovascular Diseases** | Cardiac therapy | C01 |
|  | Antihypertensives | C02 |
|  | Diuretics | C03 |
|  | Beta blocking agents | C07 |
|  | Calcium channel blockers | C08 |
|  | Agents acting on the renin-angiotensin system | C09 |
|  | Lipid modifying agents | C10 |
| **Diabetes** | Insulins | A10A |
|  | Oral antidiabetics | A10B |
|  | Combined therapy of oral antidiabetics plus insulin | A10A+A10B |
| **Other** | Drugs for acid related disorders | A02 |
|  | Vitamin B1, plain and in combination with vitamin B6 and B12 | A11D |
|  | Antithrombotic agents | B01A |
|  | Alpha-adrenoreceptor antagonists | G04CA |
|  | Corticosteroids for systemic use | H02 |
|  | Antibacterials, antimycotics, antivirals for systemic use | J01, J02, J05 |
|  | Antineoplastic agents | L01 |
|  | Immunosuppressants | L04 |
|  | Antigout preparations | M04A |
