# Supplementary material for: Drug utilization in patients starting haemodialysis with a focus on cardiovascular and antidiabetic medications: an epidemiological study in the Lazio region (Italy), 2016–2020
Source: BMC Nephrol. 2024 Mar 16;25:98. doi: 10.1186/s12882-024-03539-5 (PMC10943891; doi:10.1186/s12882-024-03539-5)
Supplement: Supplementary file 3 — Supplementary Material 3 [file 12882_2024_3539_MOESM3_ESM.docx]

Additional file 3. Characteristics of the study population at start of haemodialysis by sex and age

|  | | **Sex** | | | | **Age Classes** | | | |
| --- | --- | --- | --- | --- | --- | --- | --- | --- | --- |
|  |  | **Male** | | **Female** | | **18-64** | | **65+** | |
|  |  | **N** | ***%*** | **N** | ***%*** | **N** | ***%*** | **N** | ***%*** |
| **Total** | | 2588 | *66.7* | 1294 | *33.3* | 1459 | *37.6* | 2423 | *62.4* |
| **Sex** | |  |  |  |  |  |  |  |  |
|  | **Male** |  |  |  |  | 942 | *64.6* | 1646 | *67.9* |
|  | **Female** |  |  |  |  | 517 | *35.4* | 777 | *32.1* |
| **Age** | |  |  |  |  |  |  |  |  |
|  | **18-64 years** | 942 | *36.4* | 517 | *40.0* |  |  |  |  |
|  | **65+ years** | 1646 | *63.6* | 777 | *60.0* |  |  |  |  |
| **Comorbidities** | |  |  |  |  |  |  |  |  |
|  | **Type 2 diabetes** | 434 | *16.8* | 202 | *15.6* | 160 | *11.0* | 464 | *19.1* |
|  | **Hypertensive disease** | 953 | *36.8* | 437 | *33.8* | 493 | *33.8* | 897 | *37.0* |
|  | **Anaemias (excl. acute post haemorrhagic)** | 556 | *21.5* | 338 | *26.1* | 295 | *20.2* | 599 | *24.7* |
|  | **Ischemic heart disease** | 408 | *15.8* | 94 | *7.3* | 108 | *7.4* | 394 | *16.3* |
|  | **Heart failure** | 285 | *11.0* | 137 | *10.6* | 89 | *6.1* | 333 | *13.7* |
|  | **Cerebrovascular disease** | 278 | *10.7* | 106 | *8.2* | 94 | *6.4* | 290 | *12.0* |
|  | **Conduction disorders, cardiac dysrhythmias** | 227 | *8.8* | 86 | *6.6* | 42 | *2.9* | 271 | *11.2* |
|  | **Malignancies** | 219 | *8.5* | 80 | *6.2* | 68 | *4.7* | 231 | *9.5* |
|  | **Chronic obstructive pulmonary disease** | 148 | *5.7* | 56 | *4.3* | 27 | *1.9* | 177 | *7.3* |
|  | **Respiratory failure** | 110 | *4.3* | 69 | *5.3* | 39 | *2.7* | 140 | *5.8* |
|  | **Disorders of lipoid metabolism** | 116 | *4.5* | 44 | *3.4* | 57 | *3.9* | 103 | *4.3* |
|  | **Disorders of thyroid gland** | 58 | *2.2* | 84 | *6.5* | 41 | *2.8* | 101 | *4.2* |
|  | **Overweight, obesity and other hyperalimentation (BMI>30)** | 44 | *1.7* | 44 | *3.4* | 42 | *2.9* | 46 | *1.9* |
|  | **Chronic liver disease, diseases of pancreas** | 63 | *2.4* | 24 | *1.9* | 39 | *2.7* | 48 | *2.0* |
|  | **Peptic ulcer** | 18 | *0.7* | 5 | *0.4* | 4 | *0.3* | 19 | *0.8* |
|  | **Mental disorders** | 10 | *0.4* | 9 | *0.7* | 11 | *0.8* | 8 | *0.3* |
|  | **Dementias** | 13 | *0.5* | 5 | *0.4* | 2 | *0.1* | 16 | *0.7* |
|  | **Nutritional deficiencies** | 12 | *0.5* | 4 | *0.3* | 6 | *0.4* | 10 | *0.4* |
|  | **Chronic inflammatory intestinal diseases** | 9 | *0.3* | 5 | *0.4* | 2 | *0.1* | 12 | *0.5* |
|  |  |  |  |  |  |  |  |  |  |
