# Supplementary material for: Drug utilization in patients starting haemodialysis with a focus on cardiovascular and antidiabetic medications: an epidemiological study in the Lazio region (Italy), 2016–2020
Source: BMC Nephrol. 2024 Mar 16;25:98. doi: 10.1186/s12882-024-03539-5 (PMC10943891; doi:10.1186/s12882-024-03539-5)

Additional file 4. Proportions of haemodialysis patients using study drugs in the four semesters around starting dialysis by sex


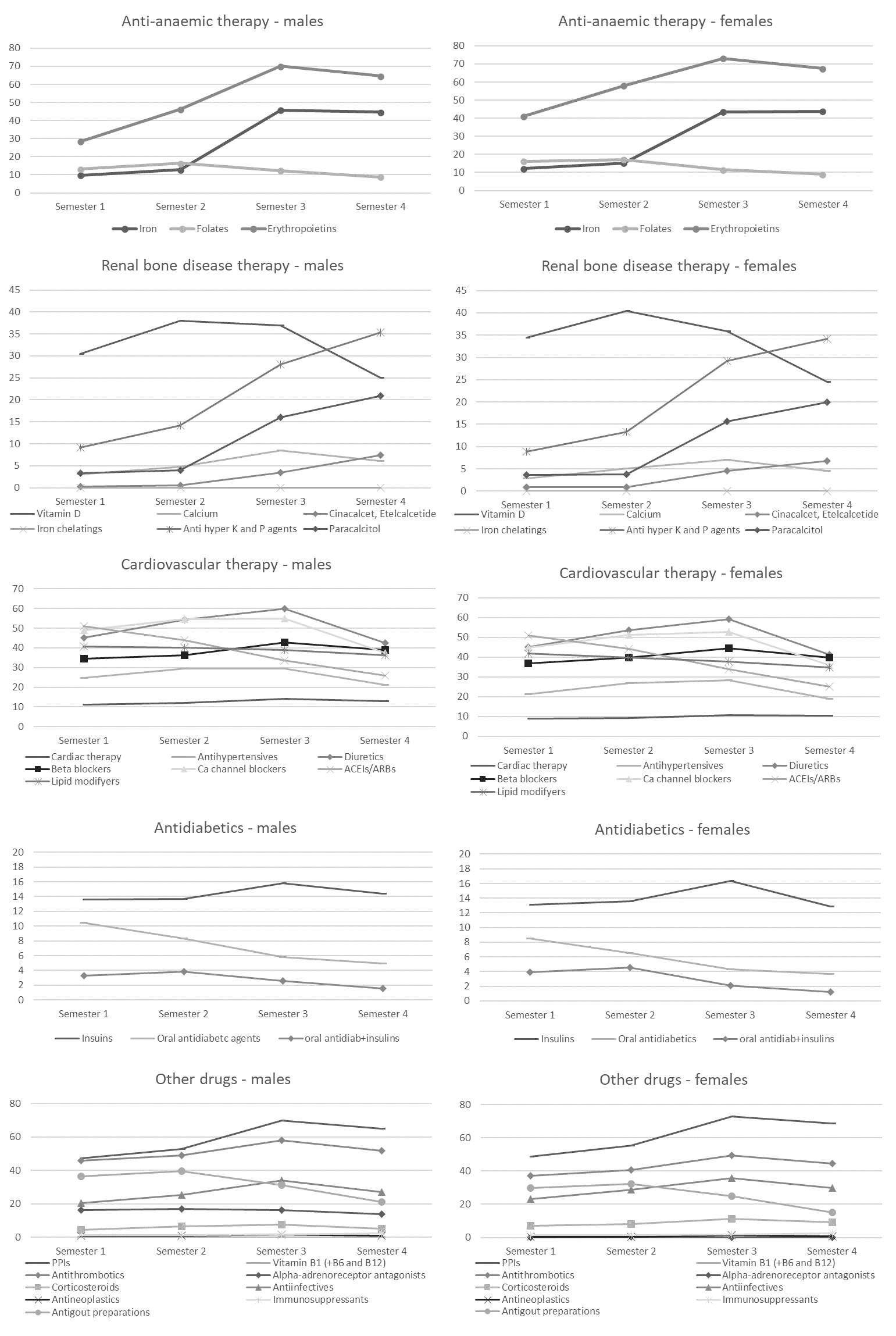

Supplement: Supplementary file 4 — Supplementary Material 4 [file 12882_2024_3539_MOESM4_ESM.docx]
