# Supplementary figures and images for: Drug utilization in patients starting haemodialysis with a focus on cardiovascular and antidiabetic medications: an epidemiological study in the Lazio region (Italy), 2016–2020
Source: BMC Nephrol. 2024 Mar 16;25:98. doi: 10.1186/s12882-024-03539-5 (PMC10943891; doi:10.1186/s12882-024-03539-5)

Additional file 6. Intensity of therapy with other cardiovascular drugs in the four semesters


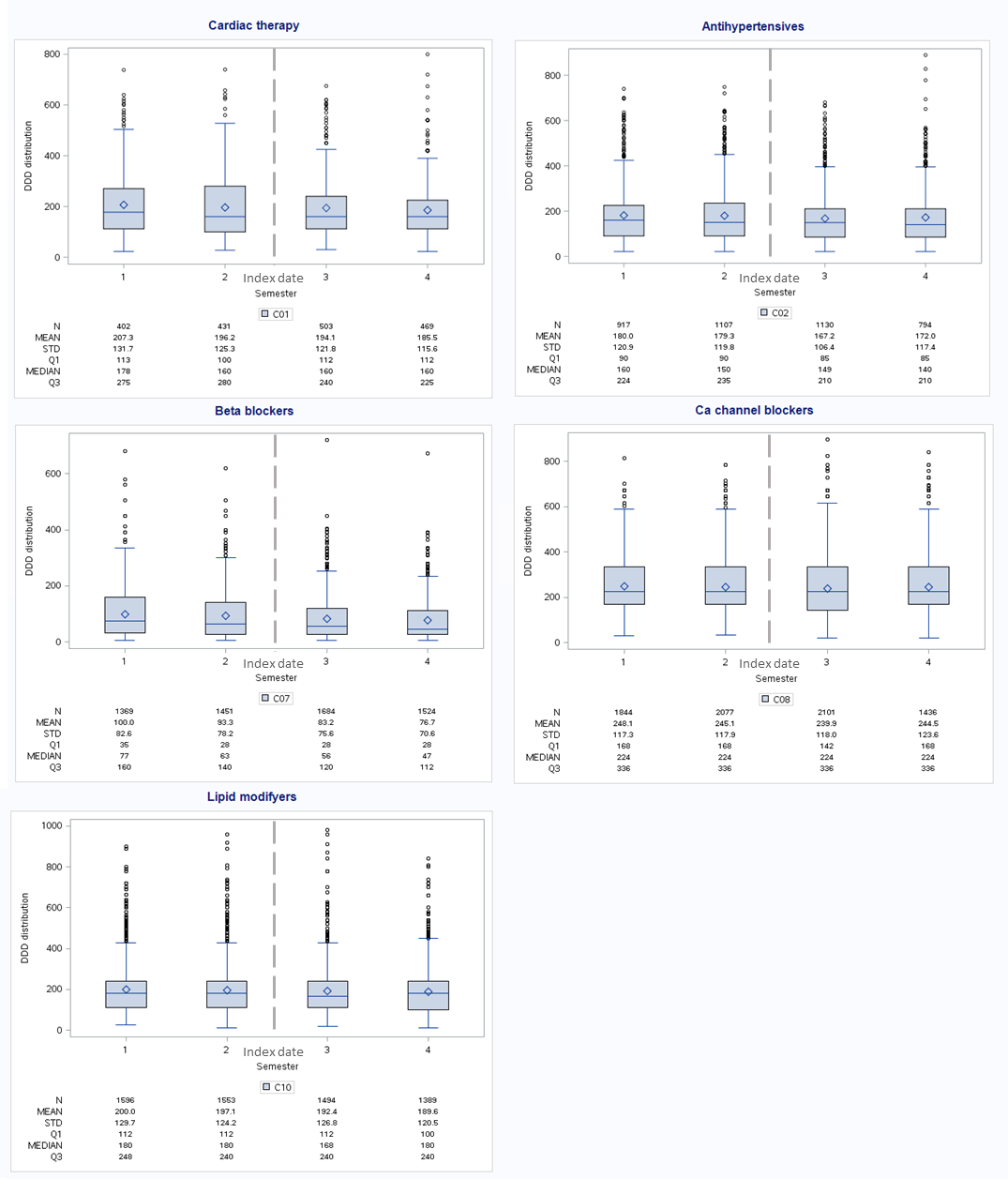

Supplement: Supplementary file 6 — Supplementary Material 6 [file 12882_2024_3539_MOESM6_ESM.docx]

Additional file 7. Intensity of therapy with cardiovascular drugs in the four semesters by sex
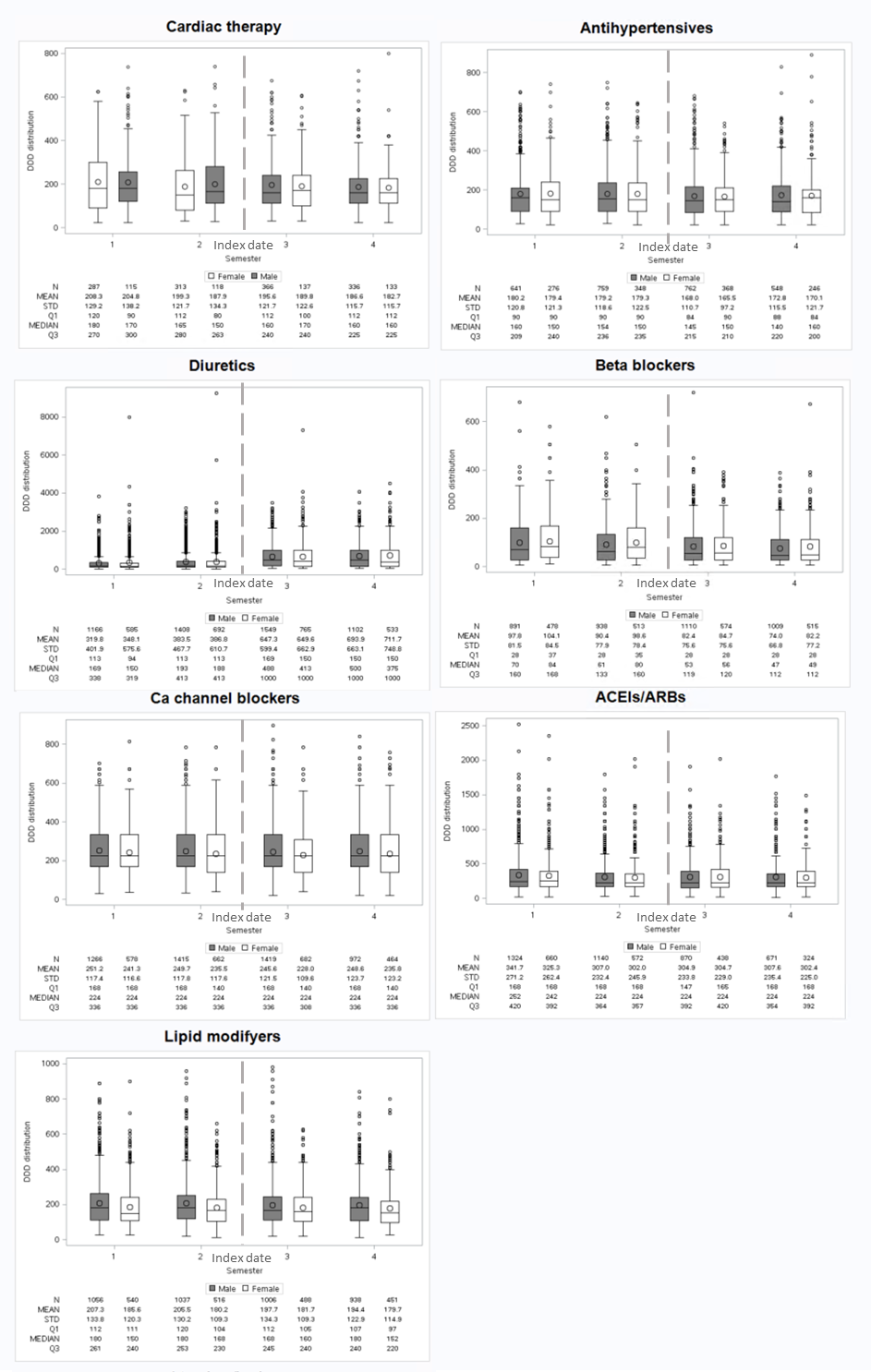

Supplement: Supplementary file 7 — Supplementary Material 7 [file 12882_2024_3539_MOESM7_ESM.docx]

Additional file 8. Intensity of therapy with cardiovascular drugs in the four semesters by age


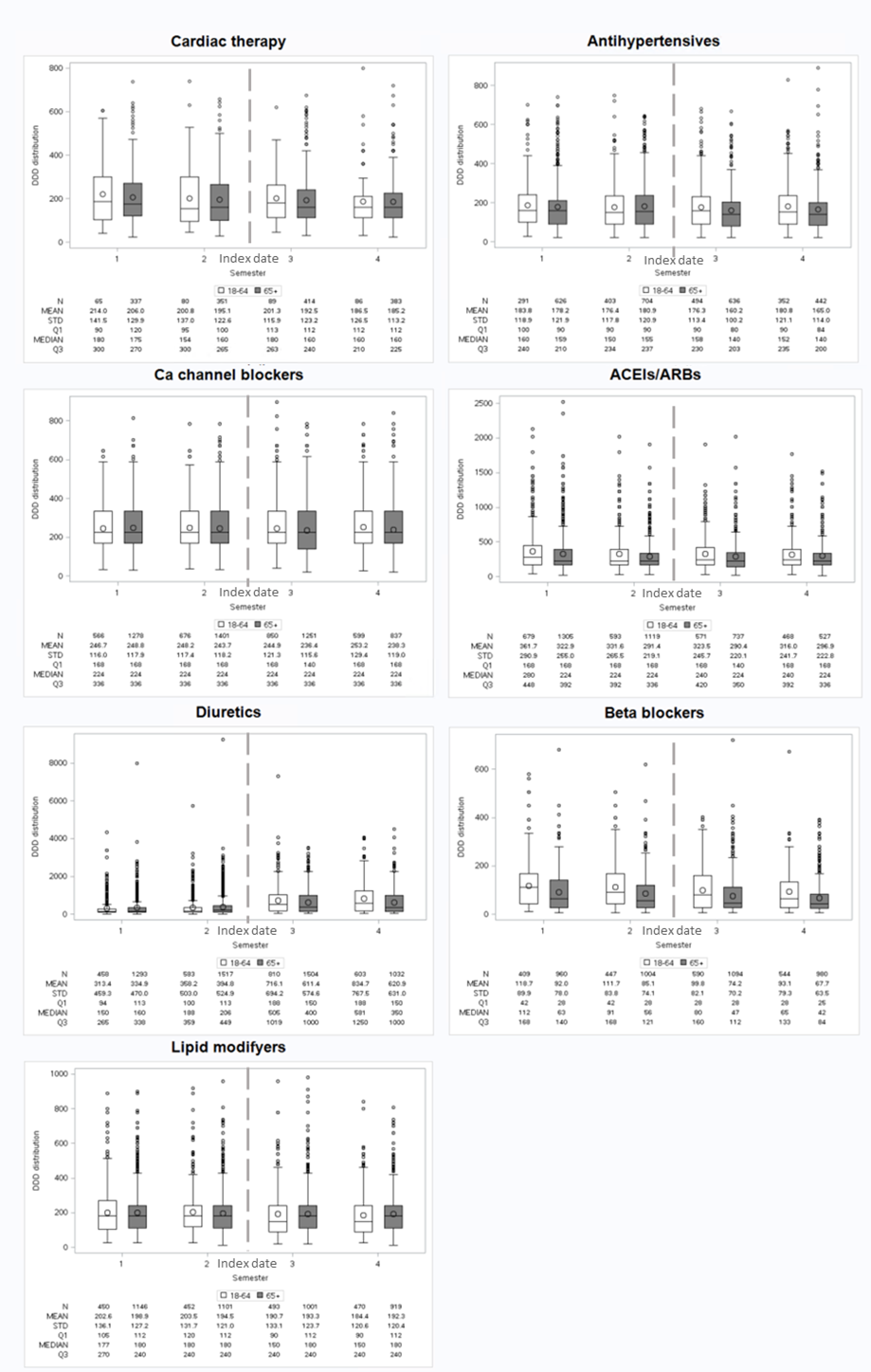

Supplement: Supplementary file 8 — Supplementary Material 8 [file 12882_2024_3539_MOESM8_ESM.docx]
